# Supplementary figures and images for: Transcriptome and proteome of the corm, leaf and flower of Hypoxis hemerocallidea (African potato)
Source: PLoS One. 2021 Jul 20;16(7):e0253741. doi: 10.1371/journal.pone.0253741 (PMC8291589; doi:10.1371/journal.pone.0253741)

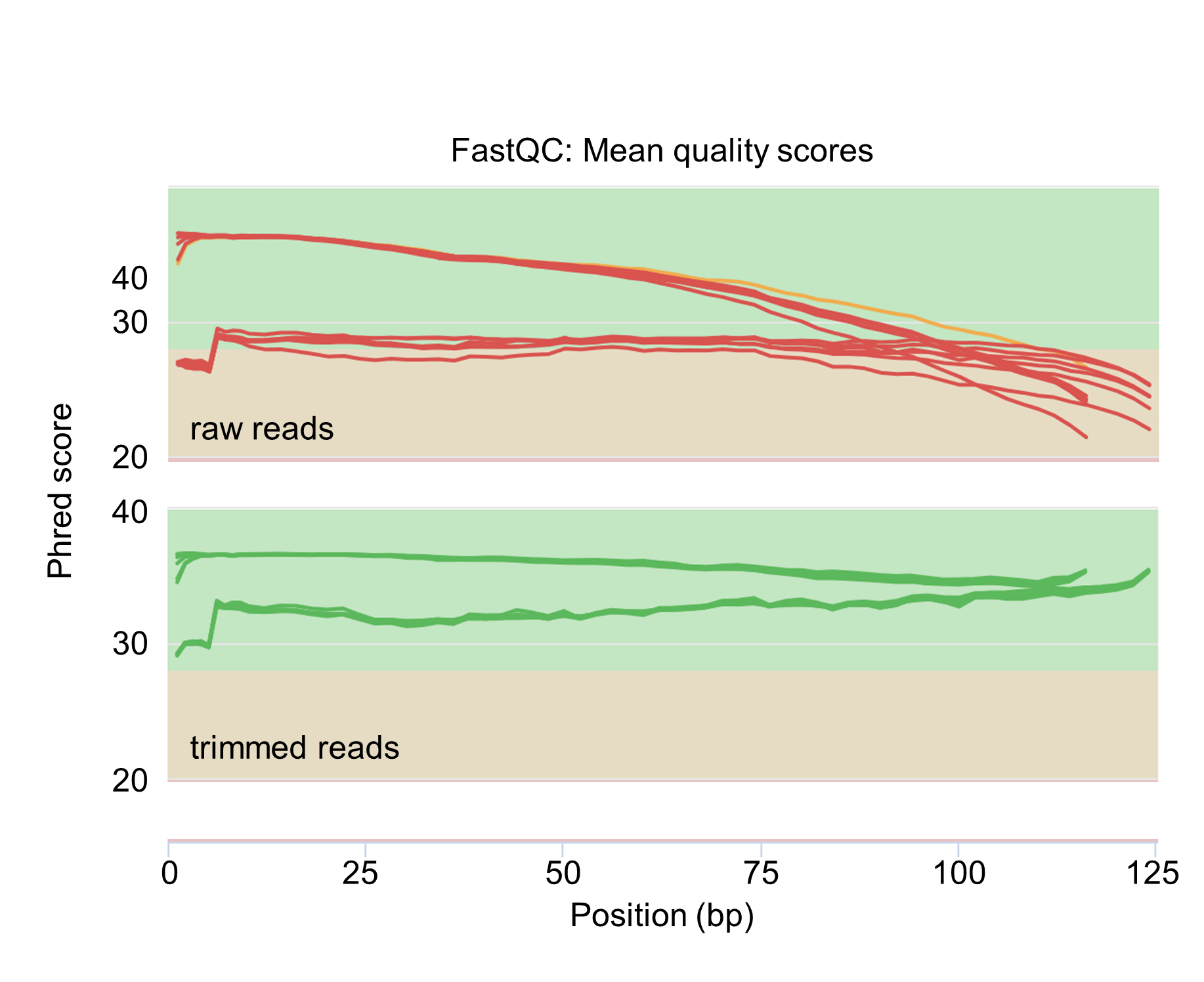

Supplement: S1 Fig — Reads were trimmed using Trimmomatic 0.36 using parameters described in the methods and materials. After trimming, more than 97% of the reads have a phred score above 30. The colour of the lines depicts the quality of the reads. Green indicates good quality; orange indicates medium quality and red indicates low quality. (TIF) [file pone.0253741.s001.tif]

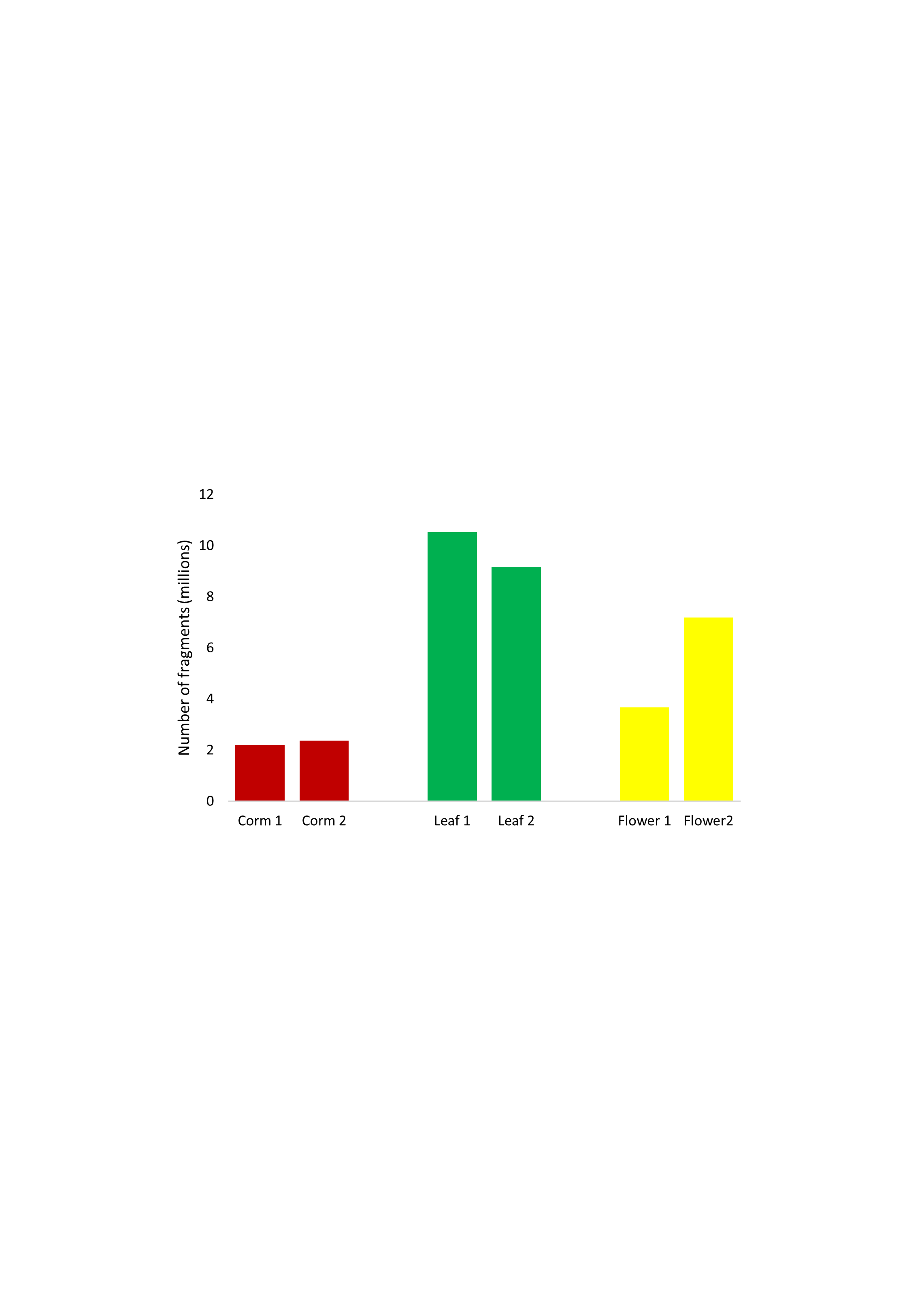

Supplement: S2 Fig — The total number of fragments is 35,087,914 between all sample replicates. (TIF) [file pone.0253741.s002.tif]

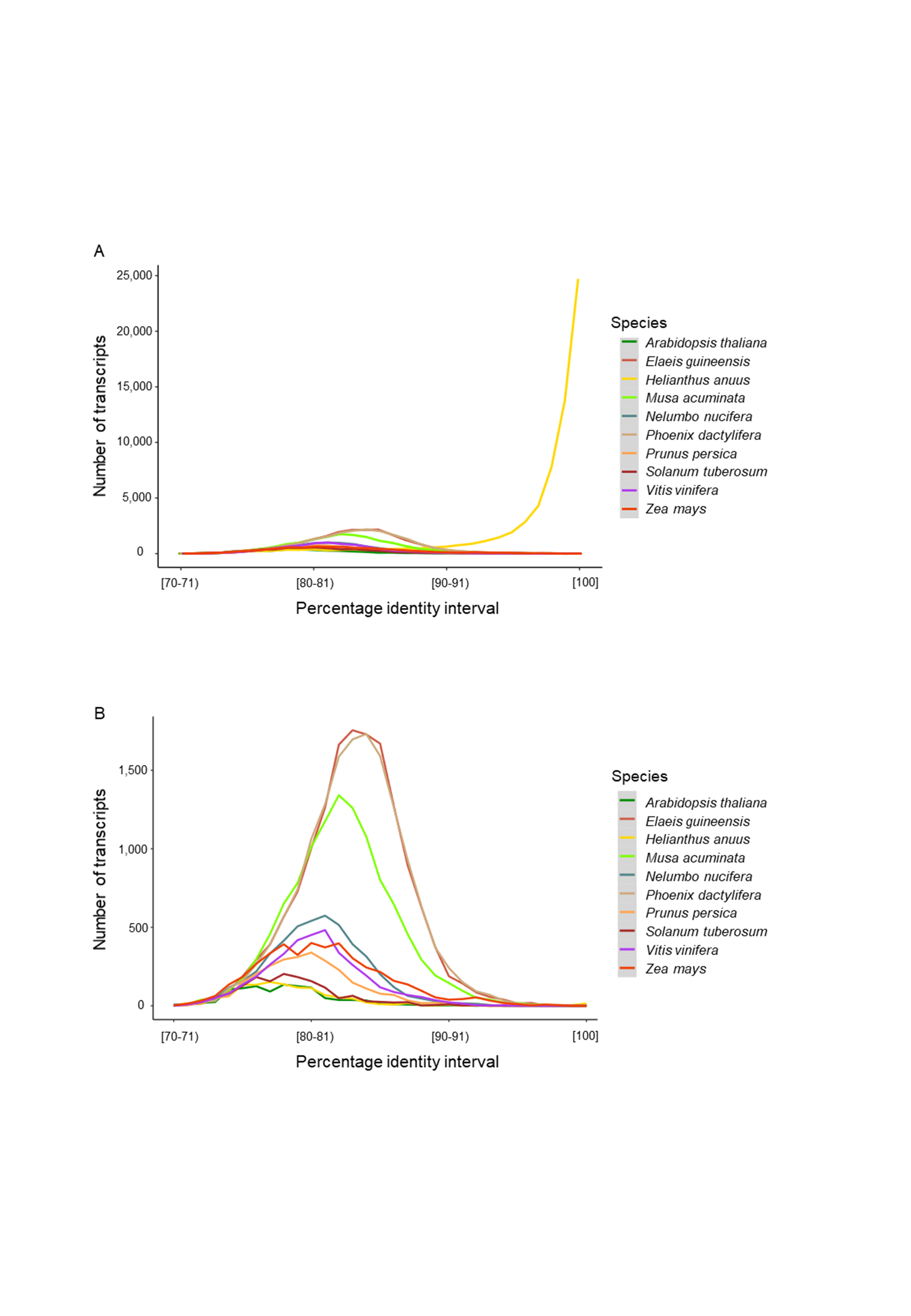

Supplement: S3 Fig — (A) Before removal of Helianthus annuus contaminating sequences originating from the multiplex experiment setup and (B) after removal of contaminating sequences with DeconSeq. (TIF) [file pone.0253741.s003.tif]

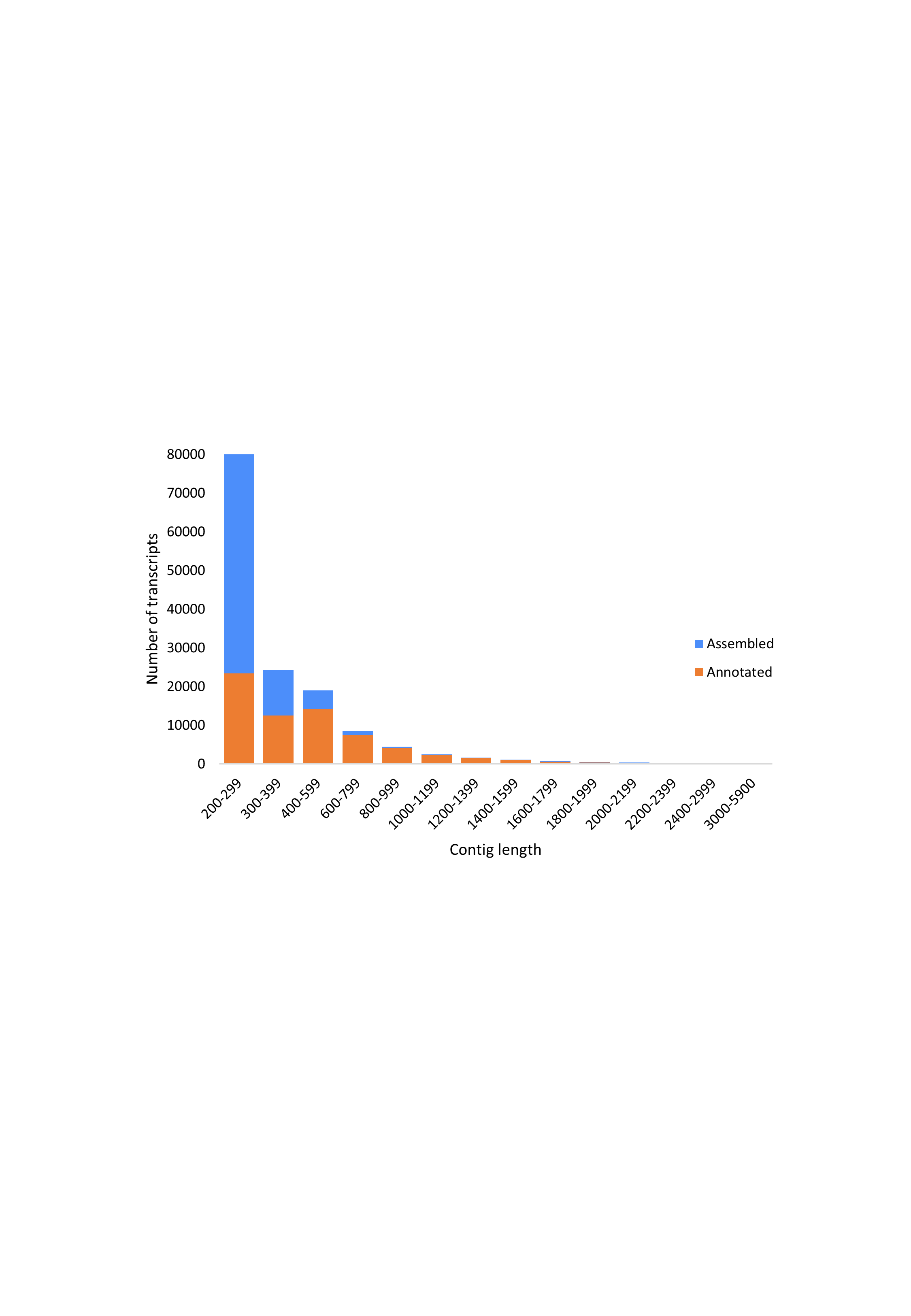

Supplement: S4 Fig — Transcripts annotated between the COG, KEGG, nr and Swiss-Prot databases with a cut-off e-value of 1e-5 are coloured in orange. A majority portion of the transcripts with a length below 300 nucleotides were not annotated (57,287 of 80,726 transcripts shorter than 300 nucleotides, i.e. 71%). (TIF) [file pone.0253741.s004.tif]

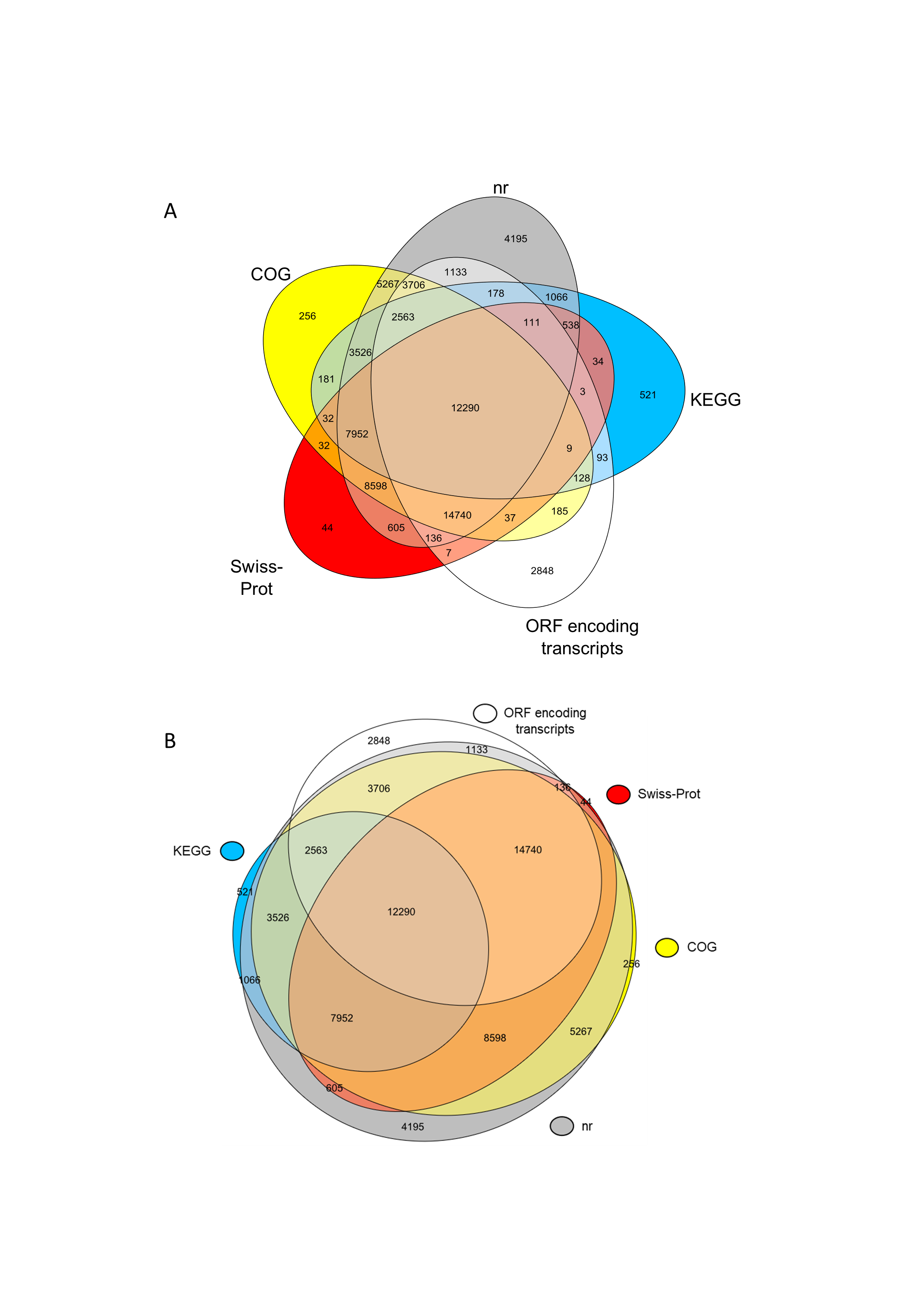

Supplement: S5 Fig — A) Venn diagram of the assembled transcripts annotated with an e-value cut-off of 1e-5 on the COG, KEGG, nr and Swiss-Prot databases and the transcripts encoding open reading frames (ORFs). The diagram presents the overlap of transcripts annotated between the database in a numerically accurate manner. B) Euler diagram depicting the proportional overlap between the annotation of transcript isoforms on the COG, KEGG, nr and Swiss-Prot databases as well as transcripts which were identified to encode ORFs with Transdecoder. (TIF) [file pone.0253741.s005.tif]

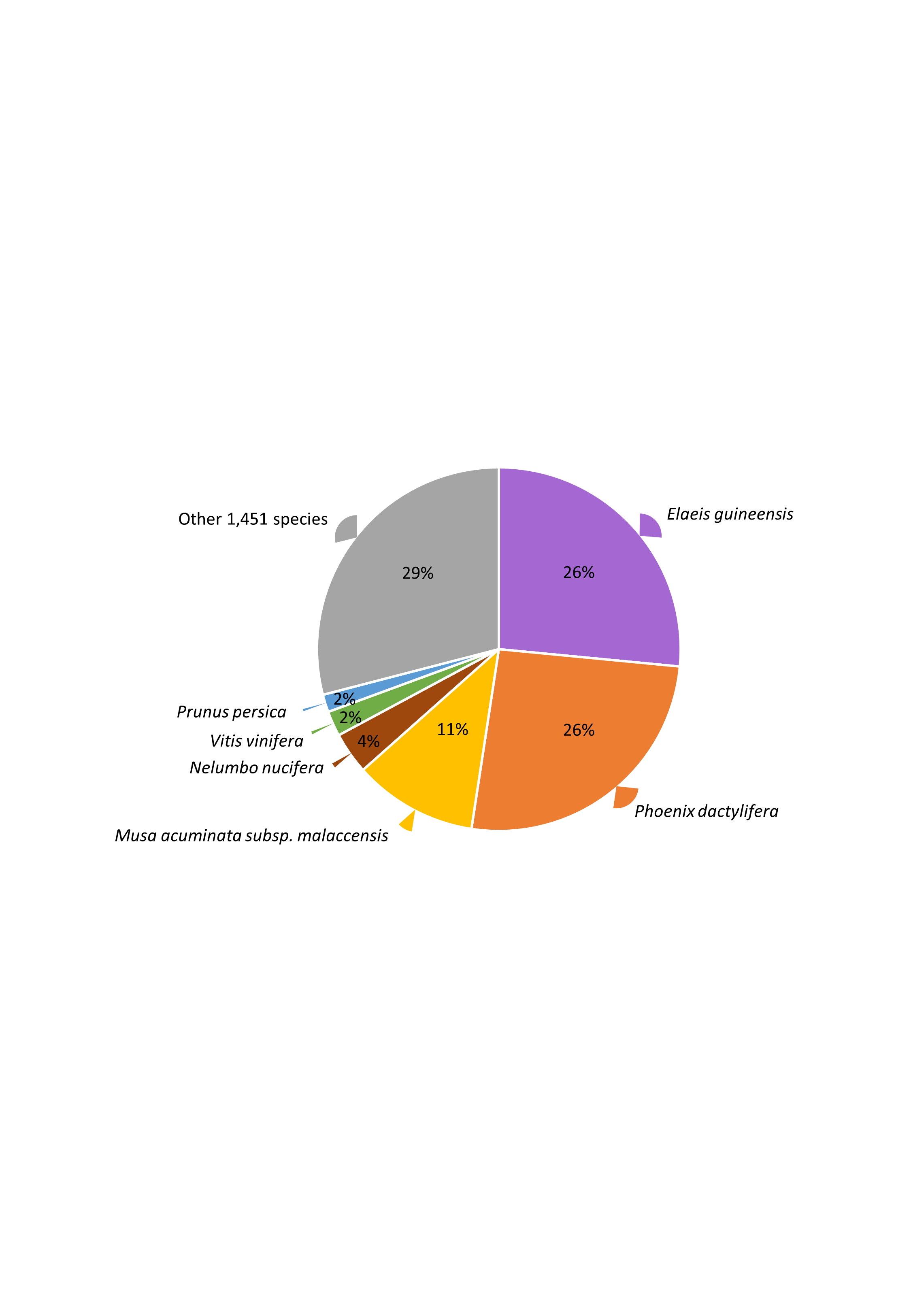

Supplement: S6 Fig — Many of the transcripts were closely associated with Elaeis guineensis (African oil palm tree) and Phoenix dactylifera (date palm). Musa acuminata subsp. Malaccensis (banana) accounts for a significant portion of the transcripts as well. The remaining transcripts were annotated amongst 1,451 species. (TIF) [file pone.0253741.s006.tif]

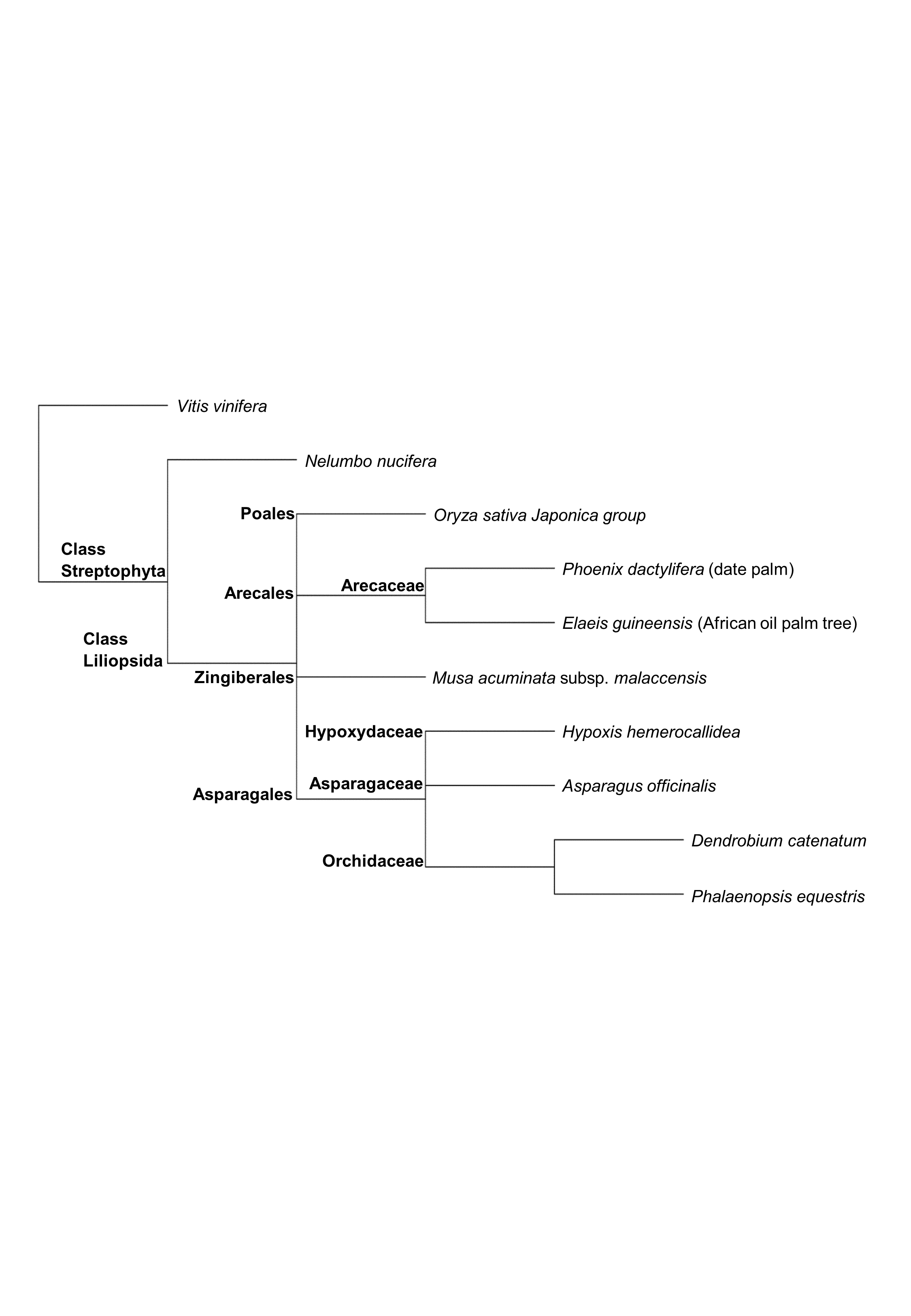

Supplement: S7 Fig — In addition, three members from the Asparagales order were also included. The order and family within class Liliopsida are labelled in bold. Species are italicised. Taxonomic lineages were identified on NCBI Common Tree. (TIF) [file pone.0253741.s007.tif]

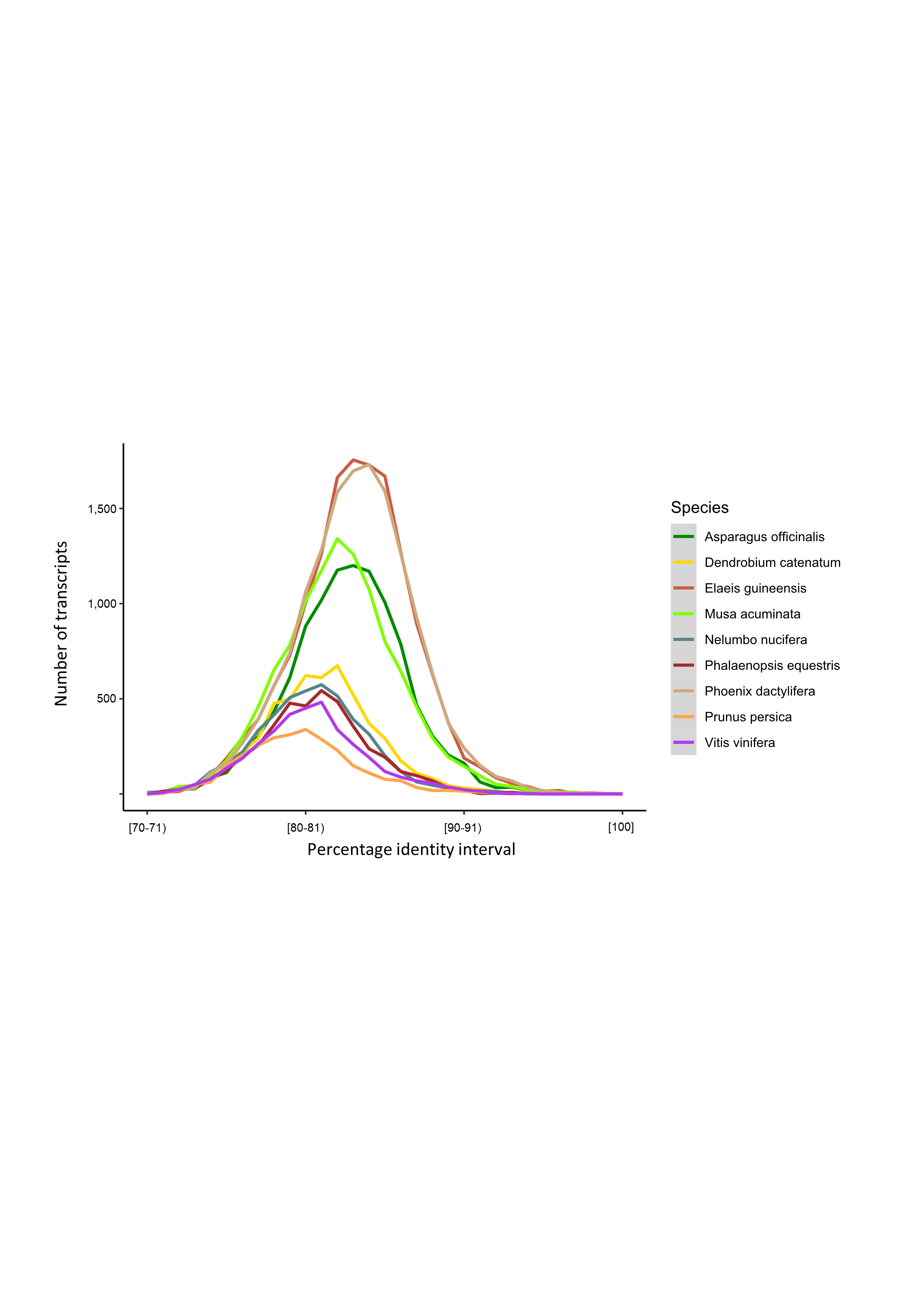

Supplement: S8 Fig — Asparagales officinalis, Dendrobium catenatum and Phalaenopsis equestris belonging to the Asparagales order are included as well. (TIF) [file pone.0253741.s008.tif]

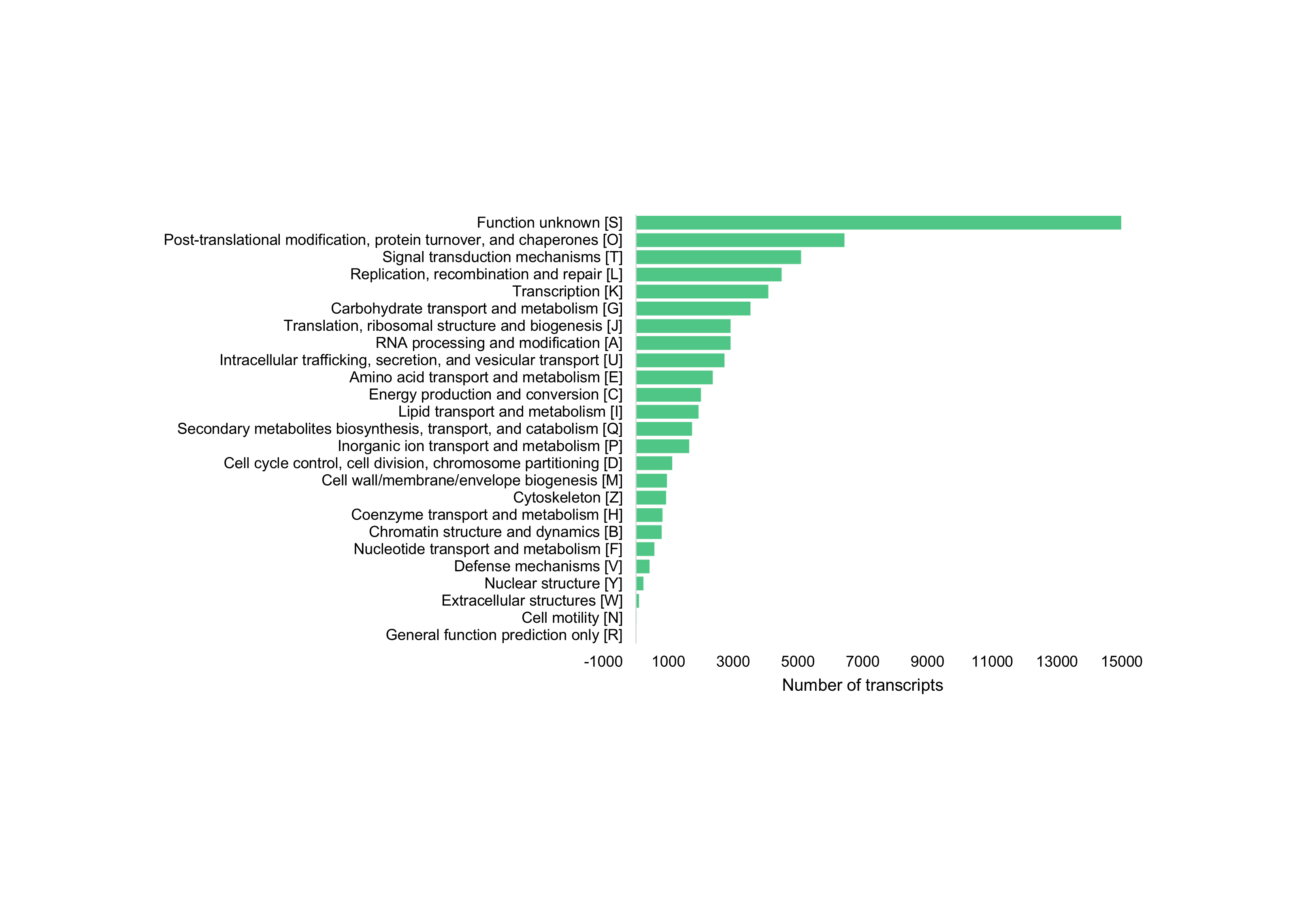

Supplement: S9 Fig — (TIF) [file pone.0253741.s009.tif]

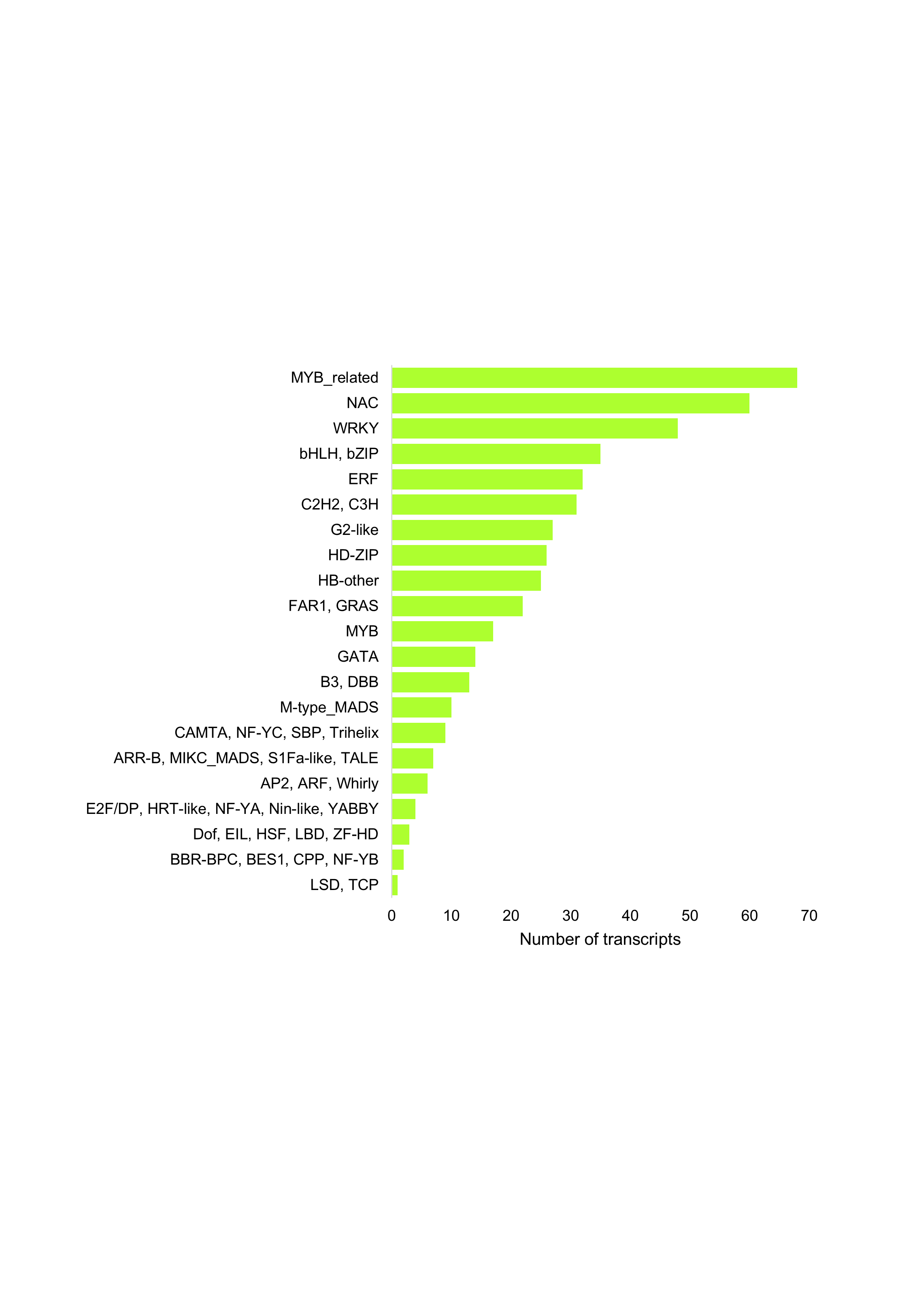

Supplement: S10 Fig — (TIF) [file pone.0253741.s010.tif]

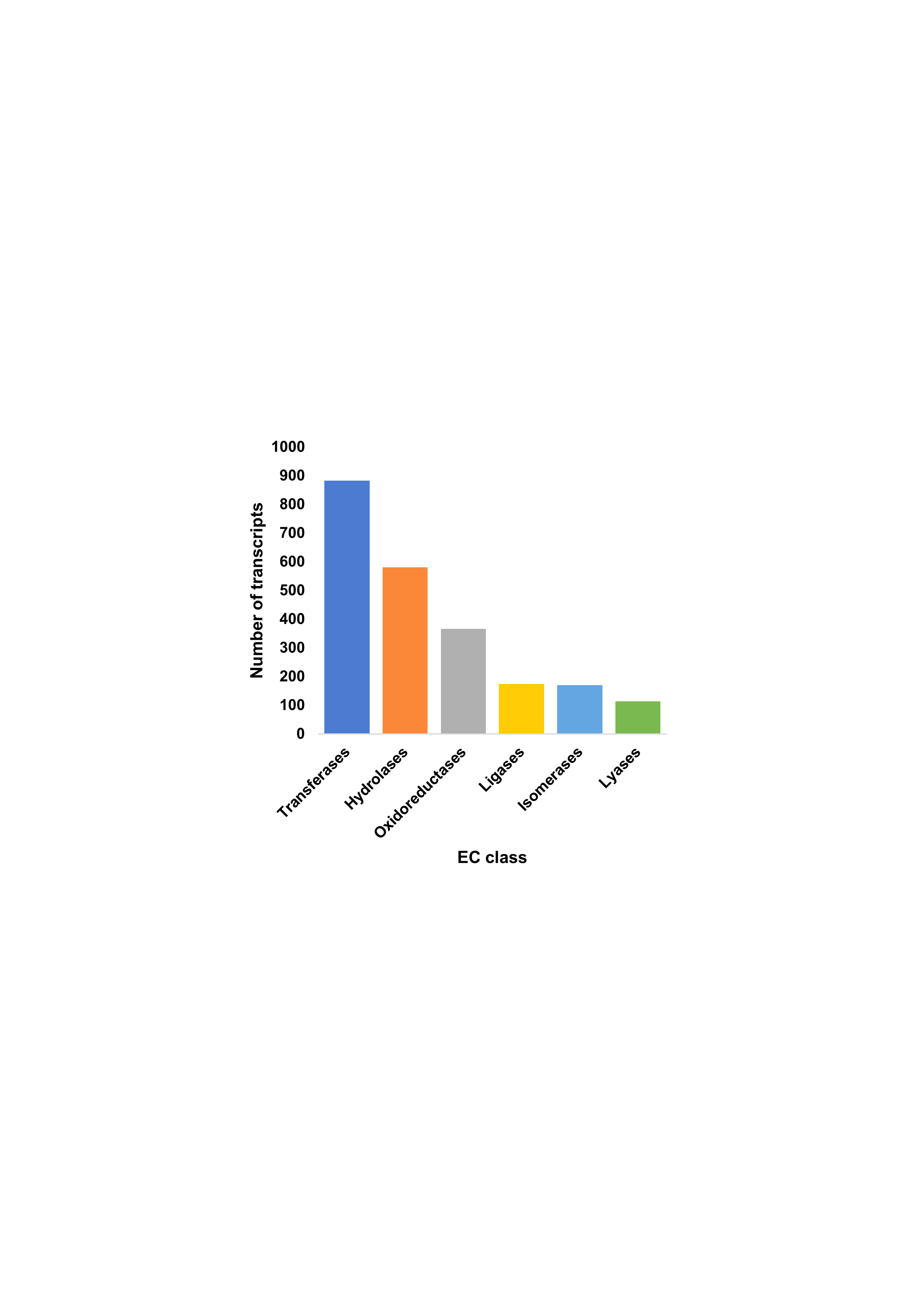

Supplement: S11 Fig — (TIF) [file pone.0253741.s011.tif]

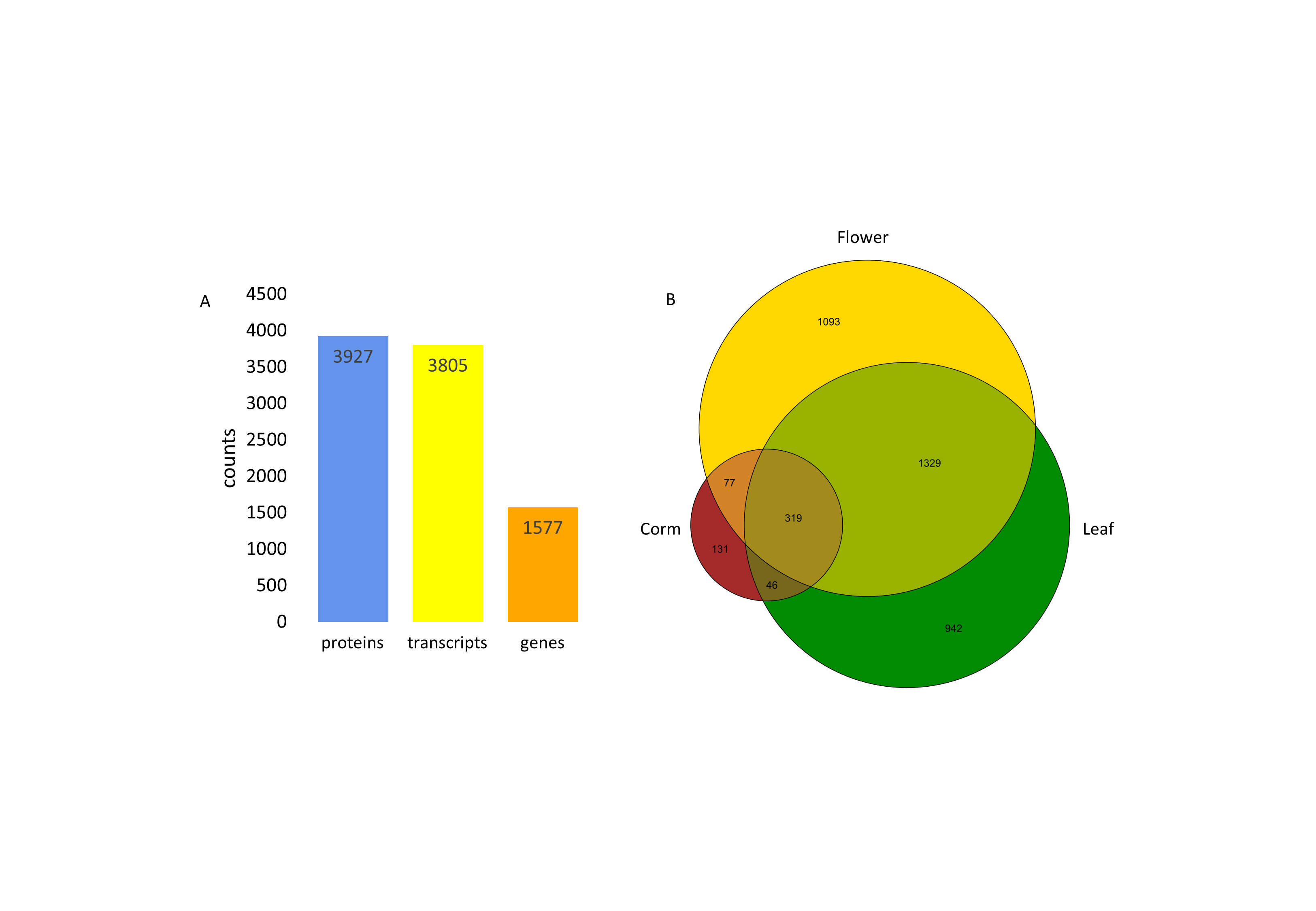

Supplement: S12 Fig — A) A total of 3,927 proteins were identified translated from 3,805 transcripts transcribed from 1,577 genes. B) The flower tissue yielded the largest number of proteins between the three tissues. (TIF) [file pone.0253741.s012.tif]

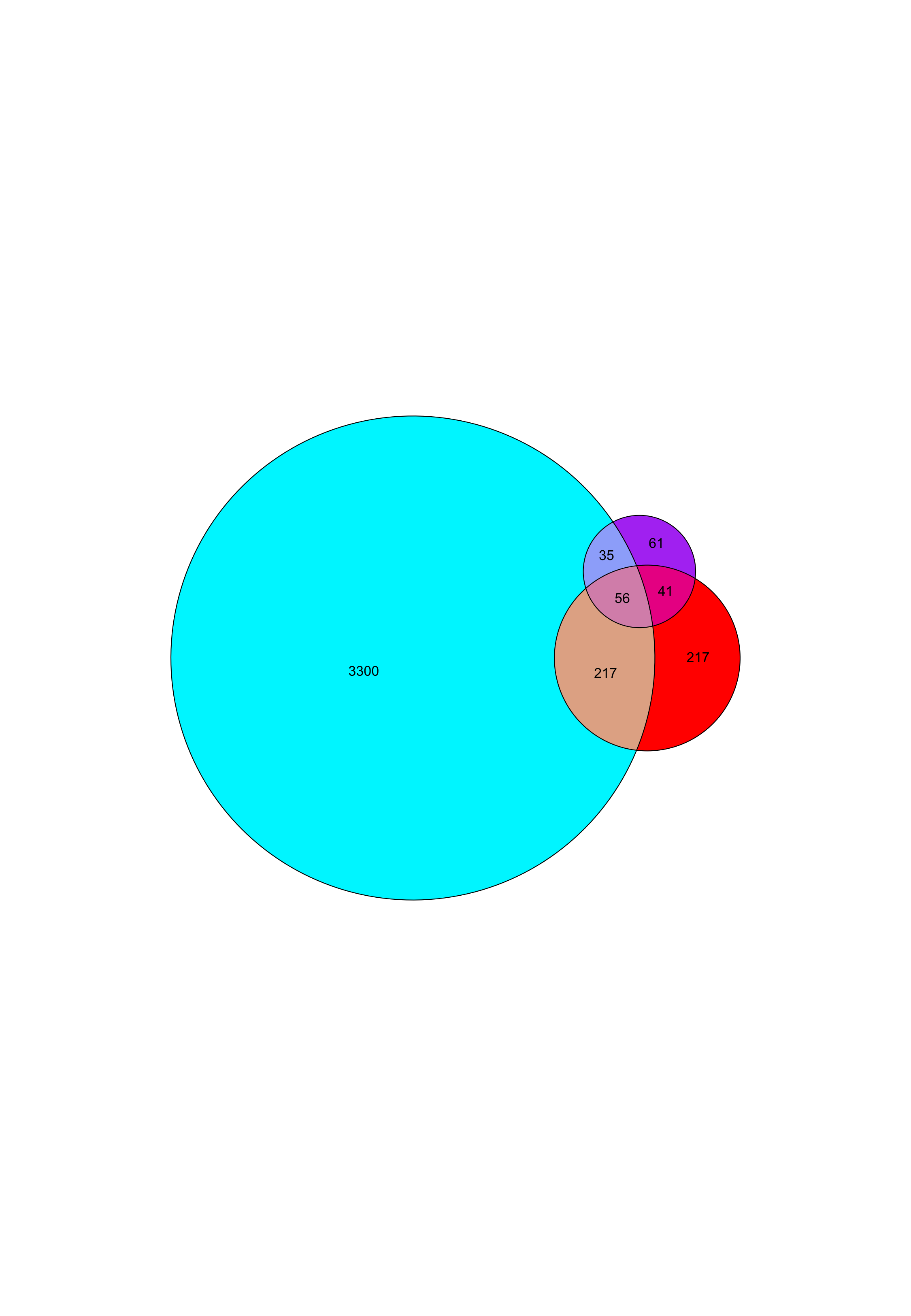

Supplement: S13 Fig — The extraction methodologies used are, SDS extraction, P-PER (ThermofischerScientific®) and fractionation of soluble corm proteins. (TIF) [file pone.0253741.s013.tif]

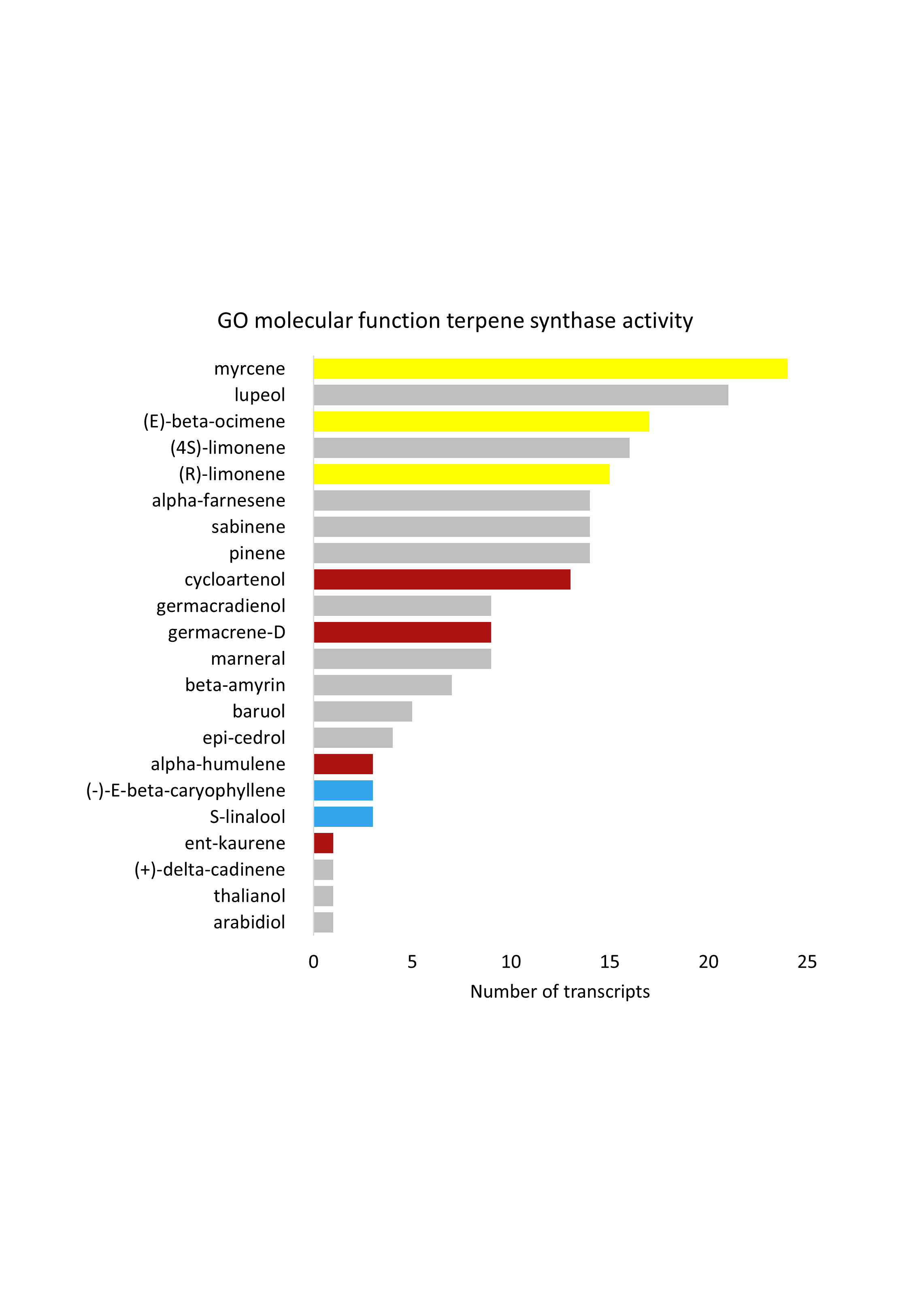

Supplement: S14 Fig — Yellow–terpene synthases that although annotated by nr, Mercator4 and GO, function could +only be retrieved from GO annotation. Grey–nr annotated terpene synthases attributed with multiple terpene synthase activity by GO. Brown–GO terpene synthase activity corresponding to the terpene synthases mapped by Mercator 4 and nr. Light blue–terpene synthases annotated by Go and nr but not Mecator4. (TIF) [file pone.0253741.s014.tif]

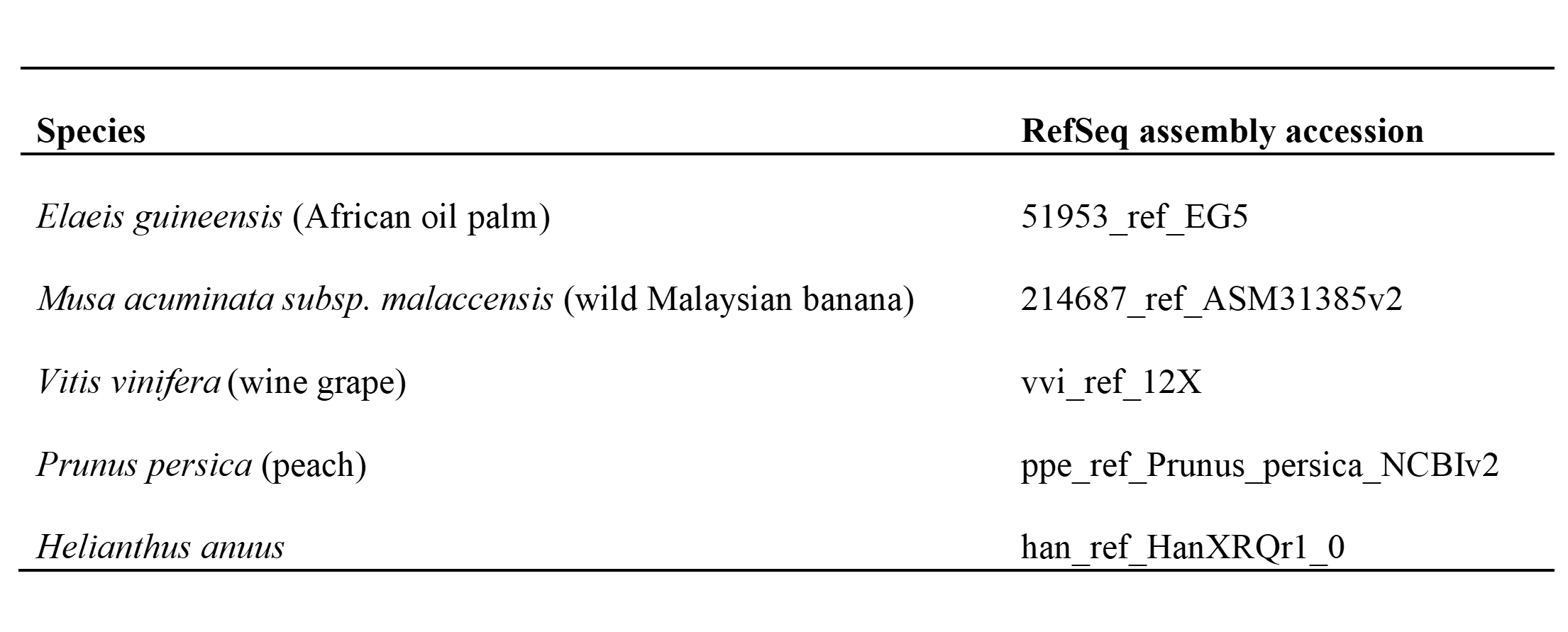

Supplement: S1 Table — (TIF) [file pone.0253741.s015.tif]

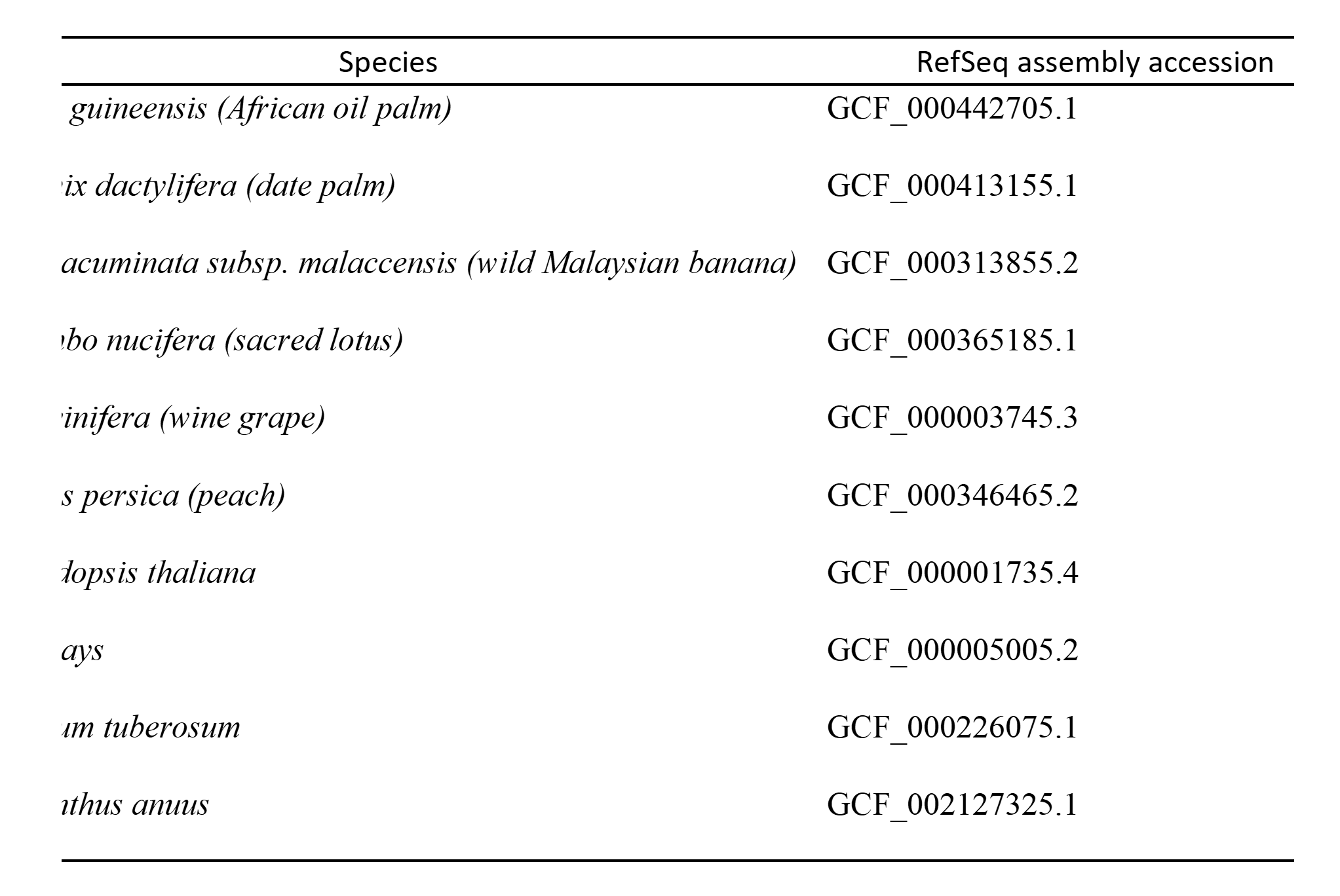

Supplement: S2 Table — Helianthuus anuus was included to compare and assess decontamination efficacy. (TIF) [file pone.0253741.s016.tif]
