# Supplementary material for: Transcriptome and proteome of the corm, leaf and flower of Hypoxis hemerocallidea (African potato)
Source: PLoS One. 2021 Jul 20;16(7):e0253741. doi: 10.1371/journal.pone.0253741 (PMC8291589; doi:10.1371/journal.pone.0253741)
Supplement: Attachment — Submitted filename: Response to Reviewers.docx [file pone.0253741.s018.docx]

# Response to Reviewers Journal Requirements:

When submitting your revision, we need you to address these additional requirements.

1. Please ensure that your manuscript meets PLOS ONE's style requirements, including those for file naming. The PLOS ONE style templates can be found at

<https://journals.plos.org/plosone/s/file?id=wjVg/PLOSOne_formatting_sample_main_body.pdf> and

<https://journals.plos.org/plosone/s/file?id=ba62/PLOSOne_formatting_sample_title_authors_affiliations.pdf>

2.Thank you for stating the following in the Financial Disclosure section:

[Our gratitude goes to the Department of Science and Technology, South Africa, for the Biocatalysis Initiative. MST received a PhD bursary from the National Research Foundation, South Africa, grant number ].

We note that one or more of the authors are employed by a commercial company: Omics Center Graz, BioTechMed and ACIB GmbH

1. Please provide an amended Funding Statement declaring this commercial affiliation, as well as a statement regarding the Role of Funders in your study. If the funding organization did not play a role in the study design, data collection and analysis, decision to publish, or preparation of the manuscript and only provided financial support in the form of authors' salaries and/or research materials, please review your statements relating to the author contributions, and ensure you have specifically and accurately indicated the role(s) that these authors had in your study. You can update author roles in the Author Contributions section of the online submission form.

Please also include the following statement within your amended Funding Statement.

“The funder provided support in the form of salaries for authors [insert relevant initials], but did not have any additional role in the study design, data collection and analysis, decision to publish, or preparation of the manuscript. The specific roles of these authors are articulated in the ‘author contributions’ section.”

If your commercial affiliation did play a role in your study, please state and explain this role within your updated Funding Statement.

2. Please also provide an updated Competing Interests Statement declaring this commercial affiliation along with any other relevant declarations relating to employment, consultancy, patents, products in development, or marketed products, etc.

Within your Competing Interests Statement, please confirm that this commercial affiliation does not alter your adherence to all PLOS ONE policies on sharing data and materials by including the following statement: "This does not alter our adherence to  PLOS ONE policies on sharing data and materials.” (as detailed online in our guide for authors http://journals.plos.org/plosone/s/competing-interests) . If this adherence statement is not accurate and  there are restrictions on sharing of data and/or materials, please state these. Please note that we cannot proceed with consideration of your article until this information has been declared.

Please include both an updated Funding Statement and Competing Interests Statement in your cover letter. We will change the online submission form on your behalf.

 Sections for Financial Disclosure, Funding Statement and Competing Interests Statement have been included in the cover letter.

Please know it is PLOS ONE policy for corresponding authors to declare, on behalf of all authors, all potential competing interests for the purposes of transparency. PLOS defines a competing interest as anything that interferes with, or could reasonably be perceived as interfering with, the full and objective presentation, peer review, editorial decision-making, or publication of research or non-research articles submitted to one of the journals. Competing interests can be financial or non-financial, professional, or personal. Competing interests can arise in relationship to an organization or another person. Please follow this link to our website for more details on competing interests: http://journals.plos.org/plosone/s/competing-interests

3. We note that you have included the phrase “data not shown” in your manuscript. Unfortunately, this does not meet our data sharing requirements. PLOS does not permit references to inaccessible data. We require that authors provide all relevant data within the paper, Supporting Information files, or in an acceptable, public repository. Please add a citation to support this phrase or upload the data that corresponds with these findings to a stable repository (such as Figshare or Dryad) and provide and URLs, DOIs, or accession numbers that may be used to access these data. Or, if the data are not a core part of the research being presented in your study, we ask that you remove the phrase that refers to these data.

The “data not shown” statement has been replaced with “supplementary heatmap data and annotation table” since the data is provided in the respective supplementary material.

4. Please include captions for your Supporting Information files at the end of your manuscript, and update any in-text citations to match accordingly. Please see our Supporting Information guidelines for more information: http://journals.plos.org/plosone/s/supporting-information.

[Note: HTML markup is below. Please do not edit.]

Reviewers' comments:

Reviewer's Responses to Questions

**Comments to the Author**

1. Is the manuscript technically sound, and do the data support the conclusions?

The manuscript must describe a technically sound piece of scientific research with data that supports the conclusions. Experiments must have been conducted rigorously, with appropriate controls, replication, and sample sizes. The conclusions must be drawn appropriately based on the data presented.

Reviewer #1: Yes

Reviewer #2: Yes

Reviewer #3: Yes

2. Has the statistical analysis been performed appropriately and rigorously?

Reviewer #1: No

Reviewer #2: Yes

Reviewer #3: Yes

3. Have the authors made all data underlying the findings in their manuscript fully available?

The [PLOS Data policy](http://www.plosone.org/static/policies.action#sharing) requires authors to make all data underlying the findings described in their manuscript fully available without restriction, with rare exception (please refer to the Data Availability Statement in the manuscript PDF file). The data should be provided as part of the manuscript or its supporting information, or deposited to a public repository. For example, in addition to summary statistics, the data points behind means, medians and variance measures should be available. If there are restrictions on publicly sharing data—e.g. participant privacy or use of data from a third party—those must be specified.

Reviewer #1: Yes

Reviewer #2: Yes

Reviewer #3: Yes

4. Is the manuscript presented in an intelligible fashion and written in standard English?

PLOS ONE does not copyedit accepted manuscripts, so the language in submitted articles must be clear, correct, and unambiguous. Any typographical or grammatical errors should be corrected at revision, so please note any specific errors here.

Reviewer #1: Yes

Reviewer #2: Yes

Reviewer #3: Yes

5. Review Comments to the Author

Please use the space provided to explain your answers to the questions above. You may also include additional comments for the author, including concerns about dual publication, research ethics, or publication ethics. (Please upload your review as an attachment if it exceeds 20,000 characters)

Reviewer #1: The research question is well defined, and I believe the experiment was well designed and executed properly. However, there are a few issues, as highlighted in the basic reporting section, where some information should be clarified, or extra information should be included. In particular, please be more transparent and replicates, comparisons, and use of the same/different tissue for the qPCR.
Validity of the findings
I believe this experiment to have been executed rigorously, but the manuscript needs more detail to ensure transparency about the number of plants/replicates/trials done.

1 The differential expressed genes must be validated using qRT-PCR, please added this part correctly. Because I noticed that the author only used two replicates for transcription sequencing.

RNA-seq has become an extremely efficient technology for quantifying relative transcript abundance and studies have shown that its accuracy rivals that of other methods such as microarrays and qRT-PCR, thus precluding the need for further validation (please see Su et al, 2014 below).

Nevertheless, in this study, validation was carried out at a proteomic level. We identified 328 proteins out of the 956 upregulated transcripts. This represents validation at a much larger scale than qRT-PCR can be performed at. Unfortunately, we are unable to perform additional qRT-PCR experiments at this point due to limited resources. However, we believe that these additional experiments will not add any value to this study given that proteomic validation was already performed.

Su, Z., Łabaj, P. P., Li, S., Thierry-Mieg, J., Thierry-Mieg, D., Shi, W., ... & Jones, W. D. (2014). A comprehensive assessment of RNA-seq accuracy, reproducibility and information content by the Sequencing Quality Control Consortium. ***Nature Biotechnology***, 32(9), 903-914

2 Association analyzed must be conducted between the differential expressed genes (DEGs) and the differential expressed proteins (DEPs) identified in your study. Positive? Negative or neither? Please added them and make it clearly.

The differentially expressed transcripts were validated using proteomic analysis. However, the proteomic analysis study was not carried out quantitatively to compare differential protein expression with differential transcript expression. Rather, it was performed to confirm that a large portion of the upregulated transcripts were also found within the proteome.

This information was discussed in lines 708 to 716 in the Revised manuscript with Track Changes:

“Having identified 3,927 unique proteins and 143,549 transcripts across the three tissues, this producing a proportion of one protein to every ~36.6 transcripts (or 2.7%) at a global level. However, this ratio is much higher when comparing the number of proteins identified within the upregulated transcripts with the number of upregulated transcripts. That is, 328 protein matches out of 956 upregulated transcripts resulting in one protein for every ~2.9 upregulated transcripts (or 34.7%) (Figs 3 and 4). This indicates that the proteomic profiling is reflective of the upregulated transcripts identified within the 3 tissues and serves to validate to a large extent the differentially expressed transcripts identified in Fig 4.”

Basic Reporting
Abstract and Introduction
Overall the information presented in the abstract and introduction is relevant and interesting and does a good job at describing the background and the biological question. However, the English is not very clear in places and therefore the manuscript would benefit from proofreading and correcting in places.
For example:

1 • Line 5-7: “The metabolites of H. hemerocallidea have been identified in several studies. More recently, the terpenoids of the plant have been identified .However, the biochemical pathways and the enzymes involved in the production of metabolites have not been characterised..”
This sentence is incompletely and needed to make clearly.

Line 5-7 has been rephrased to make the meaning clear.

The sentences now read as:

‘The metabolites contributing to the medicinal properties of *H. hemerocallidea* have been identified in several studies and, more recently, the active terpenoids of the plant were profiled. However, the biosynthetic pathways and the enzymes involved in the production of the terpene metabolites in *H. hemerocallidea* have not been characterised at a transcriptomic or proteomic level.’

Materials and methods
The level of detail is sufficient but the English needs revision.

1 •Line 84-85 “Flower and leaf material were immediately frozen in liquid nitrogen upon collection and stored at -80 ºC until use”.
Please add the information how to collected flower and leaf material? How many plants? How many days after planting and How to keep the plant? as well as how many replicates? Make all of them carefully and clearly.

Information on how to collect the flower and leaf material, collection time from planting (not in days as that information is not available), natural growing conditions, and whether specimens were collected as biological replicates or technical replicates was added.

The paragraph now reads as (lines 91 to 100 in Revised Manuscript with Track Changes):

“An approximately two-year-old *Hypoxis hemerocallidea* (African potato) plant, grown under natural conditions, was identified and collected at the Pretoria National Botanical Gardens (South Africa) under the expertise of Dr. Robert Archer. Biological replicates of the flower and leaf material were cut and stored in 50 ml centrifuge tubes. The flower material included the sepal and receptacle but not the stamen. The samples were immediately frozen in liquid nitrogen and stored at -80 ºC until use. The corm was washed with distilled water, cut into cubes of approximately 2 cm^3^, frozen in liquid nitrogen and stored at -80 ºC until use. The lack of *H. hemerocallidea* specimens, restricted the experimental setup to technical replicates for the corm tissue. Unless otherwise stated, plant material was routinely crushed into a fine powder in liquid nitrogen using a sterile mortar and pestle.”

2 •Line 118-120 “The high-quality paired-end reads of the flower, leaf and corm tissue of H. hemerocallidea were concatenated and assembled de novo (in the absence of a reference genome) into a single RNA-seq dataset using Trinity version 2.6.6 under default settings”
How to eliminate for mutli-duplication’s reads, how to obtain unigene, how to assemble the contigs into one transcript, you must be make it clearly.

The methodology for de novo assembly was carried out under default settings using the Trinity software. As such, it is beyond the scope of this study to explain the presence and elimination of multi-duplication reads or the production of unigenes by the Trinity software from the multiple contigs assembled by the software. These technical aspects of the Trinity software are presented in reference 24 and cannot be reviewed here because it falls outside the scope of the study.

3 In section 153 Differential transcript expression, it is not clear what comparisons have been made. Is it any comparisons were made? Also please describe the methodology in more details, particularly with respect to replicates.
please state how you did the clustering A lot more detail is needed for this part of the analysis.

A comparison between the corm, leaf and flower tissues of H. hemerocallidea was made as outlined in the Differential expression analysis section: “ Differential transcript expression analysis between the corm, leaf and flower tissues of H. hemerocallidea was performed using edgeR [40] to identify transcripts expressed in significantly elevated levels which could possibly confer some of the phytomedicinal associated with the corm and leaf tissues.”

Hierarchical clustering is a standard procedure within the Trinity workflow implemented to depict and finalise the differential expression analysis. To elaborate on that, additions were made to the methodology in the manuscript; lines 175 to 187 in the Revised Manuscript with Track Changes:

“Alignment-based isoform abundance estimation was performed using RNA-Seq by Expectation Maximization (RSEM) version 1.3.1 [42]. That is, the raw reads of each tissue were aligned with Bowtie to the assembled transcriptome. The relative abundance of transcripts in each tissue was estimated with RSEM. Differential transcript expression analysis between the corm, leaf and flower tissues of H. hemerocallidea was performed using edgeR [43] with log2(fpkm+1) normalisation. Differential expression analysis was performed to identify transcripts expressed in significantly elevated levels which could possibly confer some of the phytomedicinal properties associated with the corm and leaf tissues. The workflow was implemented using the Trinity pipeline [27] with a cut-off p-value set to 0.05. Clustering of expressed transcripts between the sample replicates from the leaf and flower and technical replicates from the corm tissue was performed hierarchically using normalised transcript expression obtained using edgeR. Transcripts are clustered together by similar expression levels.”

Results
1. 452 Differential transcript expression
The comparison of the reads to other species is not clear – it is missing from the materials and methods section. Please clarify the reason for these species being used, how they were compared.

The comment made by the reviewer is difficult to place in context because there was no mention made of different species being compared at a differential transcript expression level within the manuscript submitted for revision.

To attempt to answer the reviewer’s question, an assumption is made that the reviewer is referring to the taxonomic distribution of the annotated transcripts. The following text was added to clarify the reason for using certain species to compare the sequence similarity between species. Lines 165 to 172 in the Revised Manuscript with Track Changes:

“*Hypoxis hemerocallidea* is classified under the Asparagales order. However, there were no species from the Asparagales order present within the top 10 similar species. To get an insight into the evolutionary relatedness of H. hemerocallidea to species from the Asparagales order a blastn search was performed against the transcriptomes of Asparagus officinalis (garden asparagus) (RefSeq: GCF_001876935.1), Dendrobium catenatum (RefSeq: GCA_001605985.2 and Phalaenopsis equestris (RefSeq: GCF_001263595.1) (from the Asparagales order). The top 6 similar species were included in the analyses as well.”

2.Please add the part of “Verification of the gene expression profiles of candidate DEGs by qRT-PCR” and “Statistical analysis” In methods and results part. Please denote the association analyzed between DEGs and DEPs identified in this research.

A detailed explanation of why qRT-PCR was not performed is provided in a previous response to this reviewer (please see comment 1).

To address the second part of the reviewer’s request: “Please denote the association analyzed between DEGs and DEPs identified in this research.”:

To numerous extents, associations have been made (“denoted”) in the manuscript between differentially expressed transcripts and proteins detected within the tissues. For example, Figures 5 and 6 are clearly relating differentially expressed transcripts and expressed proteins.

Discussion
Content in the discussion is good and is a relevant discussion of the results. However, the English is poor and therefore would benefit from proofreading.

The manuscript has been proofread and edited appropriately.

Reviewer #2: Manuscript present transcriptome and the proteome sequencing of medicinal African potato (Hypoxis hemerocallidea). Numerous terpene synthases were identified through functional annotation. Differential expression analysis showed that which tissue upregulateslinalool synthase.
It is valuable, because combined transcriptome and proteome analyses gives a complete insight of genetics and biochemistry of this valuable medicinal plant.
There are some comments that must be addressed by authors before possible acceptance of the manuscript:
Abstract:
- Lines 2-7: This paragraph is belongs to Introduction. Just one line is abstract is enough to shows the importance of plant of interest.

Reviewer 1 recommended the expansion of lines 5-7 for clarity purposes. We chose to adhere to the guidelines suggested by Reviewer 1 because this also provides additional background to why this study was carried out which may be useful to some readers. Moreover, the abstract adheres to the word limit of the journal therefore removal of sentences is not necessary.

- Line 14: … such as nerolidol synthase, germacrene D synthase, ….

A comma has been added after germacrene D synthase.

Introduction:
- Line 26: Hypoxidaceae must be written in Italic.

Hypoxidaceae is a plant family name which is not italicised. (See reference below)

(For reference please see: Nelson, L. S., & Balick, M. J. (2020). Section 1. Botanical Nomenclature and Glossary of Botanical Terms. In Handbook of Poisonous and Injurious Plants (pp. 1-18). Springer, New York, NY.)

- Line 31: Please change “medical conditions” with an appropriate phrase.

“medical conditions” changed to “illnesses”

- Line 32: … some cancers, and HIV-AIDS.

A comma has been added after ‘some cancers’.

- Line 35: Reference “(Liebenberg, 1969)” is out of the instruction of journal. Please keep the same format for references.

Citation was corrected to [3] instead of “(Liebenberg, 1969)”.

- Line 36: Latter research has corroborated some of the medicinal properties (Reference??).

References 4,5 and 6 are in text following the specific examples of corroborated medicinal properties. (Lines 39 to 43 in Revised Manuscript with Track Changes).

- Line 47: please rewrite “and so have their use in the treatment of inflammation”.

The phrase “and so have their use in the treatment of inflammation” has be re-written as “alongside their use in the treatment of inflammation”. (Line 52 of Revised Manuscript with Track Changes).

- Line 51-52: please mention the reasons why “the cultivation of H. hemerocallidea is known to be problematic”??

The sentence was extended as follows to provide a reason for difficulty in cultivation: “the cultivation of H. hemerocallidea is known to be problematic due to lengthy seed dormancy”. (Line 56 of Revised Manuscript with Track Changes)

- Line 49-52: Author mentioned that (i) synthetic production of hypoxoside is difficult, (ii) tissue culture of H. hemerocallidea produces low yields of hypoxoside rendering this method impractical, and (iii) the cultivation of H. hemerocallidea is known to be problematic. With this testimonial, what solution is provided by the authors?

The sentence: “This leaves an alternative, important and yet unexplored gap in the biocatalytic production of hypoxoside, rooperol and other important metabolites which can circumvent the need for cultivation or tissue culturing of Hypoxis hemerocallidea” was placed in text in sequence after the testimonial indicated by the reviewer. The text suggests a solution that biocatalytic alternatives could be adapted to produce the metabolites of interest. (Lines 62 and 63 in the Revised Manuscript with Track Changes).

- Line 60: Please give some examples of “ other plant sources”.

The sentence was rephrased as follows: “However, this compound can be produced on an industrial scale from *Chlorophytum borivilianum*” to provide a specific example. (Line 65 in the Revised Manuscript with Track Changes).

- Line 60: highly unlikely??!

Rephrased to “unlikely” (Line 66 in the Revised Manuscript with Track Changes).

- Line 72: …. H. hemerocallidea was …

Rephrased to: “…H. hemerocallidea was…” (Line 77 in the Revised Manuscript with Track Changes).

- Introduction section needs some examples of similar researches in other medicinal plants.

To address the request of the reviewer, the following text was added in text with some editing to text already present:

Lines 81 to 86 of Revised Manuscript with Track Changes:

“Cross-tissue transcriptomic analyses have been performed in the past to identify candidate genes involved in the biosynthesis of secondary metabolites in medicinal plants like *Ferula asafoetida*, *Dysphania schraderiana* and *Salvia miltiorrhiza* [21–23]. In this study, aside from transcriptomic analyses, proteomic profiling was performed as well on the flower leaf and corm tissues from *Hypoxis hemerocallidea*.”

Methods and materials
- Line 84: Flower and leaf materials …

In response to comments from Reviewer 1, the sentence was rephrased, and more information was added. For the sentence to remain grammatically correct, ‘material’ will not be changed to ‘materials’.

The sentence now reads as:

‘Biological replicates of the flower and leaf material were cut and stored in 50 ml centrifuge tubes.’ (Line 93 and 94 in the Revised Manuscript with Track Changes).

There is no additional comments related to other sections of “Methods and materials” and I appreciate the authors’ efforts for complete writing and explanations of these sections.

Results
- Line 357: ORFs: Please mention the abbreviations in their first use in the text.

The term “Open reading frame” was mentioned in full on line164 of the Revised Manuscript with Track Changes within the methodology section for the first time and it is not needed to re-mention here.

Discussion
- Lines 633-650: Taxonomic distribution of annotated transcripts: there is no discussion for this paragraph and it seems that it is a “Results” section not “Discussion”.

The Taxonomic distribution of annotated transcripts was discussed. Please see lines 667 to 683 in the Revised Manuscript with Track Changes.

- Lines 652-665: Paragraph “Differential expression”. It also similar to “Results” section not “Discussion”.

The Differential expression was discussed. Please see lines 686 to 698 in the Revised Manuscript with Track Changes.

- Lines 685-708; Paragraph “Terpenoid biosynthesis” there is no related references in this section.

There are not many studies to reference terpenoid profile of the African potato to at a specific level. However, references to multi-product terpenoid synthases has been made [55] as well as a reference to the metabolomic study of the African potato performed in [19]. (Line 735 and 740 in the Revised Manuscript with Track Changes).

Reviewer #3: The authors sequenced the transcriptome, proteome of three tissues of Hypoxis hemerocallidea (African potato), and did the assembly and functional annotation of the transcriptome, different expression analysis of the transcripts, cross-analysis of transcriptome and proteome, and analyzed the secondary metabolism.

I have the following major concerns:

1. The authors did decontamination on the contigs after the assembly. I suggest running decontamination on the fastq files before the assembly. the reads from the other species can cause assembly errors, and the contigs can contain a mix of the reads from different species. Maybe that is why the assembly is too fragmental.

The tool used in decontaminating the transcriptome assembled here was DeconSeq. It is a post-assembly decontamination tool published in a peer-reviewed journal (For reference see: Schmieder, R., & Edwards, R. (2011). Fast identification and removal of sequence contamination from genomic and metagenomic datasets. PloS one, 6(3), e17288.). The implementation of the tool was carried out as recommended. A pre-assembly decontamination procedure was not feasible due to the unavailability of the raw data of the contaminating species.

2. I don't recommend the authors run assembly by default parameters on Trinity and then stopped to refine the assembly results. The assembly results are the cornerstone of the whole project, better assembly results can avoid analysis errors. 

The major component of data refinement was decontamination which could not be avoided or performed pre-assembly to justify optimisation of the assembly process. Furthermore, the default parameters are also suggested by the creators of the Trinity assembler.

Some minor comments:
1. the last row of table 1 is not well described, especially the last item (n: 3278)

The “n:” refers to the number of BUSCO groups searched. The phrase “…after searching 3,278 BUSCO groups…” was added to line 374 in the Revised Manuscript with Track Changes for clarification.

2. for fig 3, set a title to the two plots. the plot on the right is not suitable for the line plot. because the data are independent of each other.

Figure 3 has a legend; a title will only add redundancy to the needed figure legend. Therefore, we opt for a diagram without a title. The second part of the reviewer’s comment is unclear. The expression level is dependent on the tissue type and the lines depict the expression level of all samples and tissues in for each cluster. The data is dependent. For clarity, the following sentence was added to the Fig 3 legend: “Line plots depict the expression levels of transcripts for each of the three clusters.”

3. at line 324-325, the authors say "A total of 35,087,914 fragments (S2 Fig) were then assembled de novo with Trinity.", but the Fig S2 title says "number of trimmed fragments", it is a little confusing, I suggest changing it as "number fragments after trimmed".

The phrase “…A total of 35,087,914 fragments (S2 Fig)…” was changed to “…A total of 35,087,914 trimmed fragments (S2 Fig)…”. (Lines 354 and 355 in the Revised Manuscript with Track Changes).

4. There is a yellow line in the raw reads plot in Fig S1, while the others are red. A legend is required for these lines.

The following sentence was added to the legend of S1 Fig: “The colour of the lines depicts the quality of the reads. Green indicates good quality; orange indicates medium quality and red indicates low quality.”

5. the manuscript is a little tedious and seems missing the focus. The authors can do more analysis and discuss more on the terpenoids as mentioned in the introduction.

We believe we have derived valuable information from the analyses as it stands and we have depicted these data in an appropriate way in a single figure (Fig 6) which makes it easy for researchers to understand the findings of this study.

6. PLOS authors have the option to publish the peer review history of their article ([what does this mean?](https://journals.plos.org/plosone/s/editorial-and-peer-review-process#loc-peer-review-history)). If published, this will include your full peer review and any attached files.


If you choose “no”, your identity will remain anonymous but your review may still be made public.


**Do you want your identity to be public for this peer review?** For information about this choice, including consent withdrawal, please see our [Privacy Policy](https://www.plos.org/privacy-policy).

Reviewer #1: No

Reviewer #2: **Yes:** Mohsen Niazian

Reviewer #3: No

[NOTE: If reviewer comments were submitted as an attachment file, they will be attached to this email and accessible via the submission site. Please log into your account, locate the manuscript record, and check for the action link "View Attachments". If this link does not appear, there are no attachment files.]

While revising your submission, please upload your figure files to the Preflight Analysis and Conversion Engine (PACE) digital diagnostic tool, <https://pacev2.apexcovantage.com/>. PACE helps ensure that figures meet PLOS requirements. To use PACE, you must first register as a user. Registration is free. Then, login and navigate to the UPLOAD tab, where you will find detailed instructions on how to use the tool. If you encounter any issues or have any questions when using PACE, please email PLOS at [figures@plos.org](mailto:figures@plos.org). Please note that Supporting Information files do not need this step.
