# Supplementary material for: Transcriptome and proteome of the corm, leaf and flower of Hypoxis hemerocallidea (African potato)
Source: PLoS One. 2021 Jul 20;16(7):e0253741. doi: 10.1371/journal.pone.0253741 (PMC8291589; doi:10.1371/journal.pone.0253741)
Supplement: Attachment — Submitted filename: Response to reviewers.docx [file pone.0253741.s019.docx]

**Comments to the Author**

1. If the authors have adequately addressed your comments raised in a previous round of review and you feel that this manuscript is now acceptable for publication, you may indicate that here to bypass the “Comments to the Author” section, enter your conflict of interest statement in the “Confidential to Editor” section, and submit your "Accept" recommendation.

Reviewer #1: All comments have been addressed

Reviewer #3: (No Response)

2. Is the manuscript technically sound, and do the data support the conclusions?

The manuscript must describe a technically sound piece of scientific research with data that supports the conclusions. Experiments must have been conducted rigorously, with appropriate controls, replication, and sample sizes. The conclusions must be drawn appropriately based on the data presented.

Reviewer #1: Yes

Reviewer #3: Yes

3. Has the statistical analysis been performed appropriately and rigorously?

Reviewer #1: Yes

Reviewer #3: Yes

4. Have the authors made all data underlying the findings in their manuscript fully available?

The [PLOS Data policy](http://www.plosone.org/static/policies.action#sharing) requires authors to make all data underlying the findings described in their manuscript fully available without restriction, with rare exception (please refer to the Data Availability Statement in the manuscript PDF file). The data should be provided as part of the manuscript or its supporting information, or deposited to a public repository. For example, in addition to summary statistics, the data points behind means, medians and variance measures should be available. If there are restrictions on publicly sharing data—e.g. participant privacy or use of data from a third party—those must be specified.

Reviewer #1: Yes

Reviewer #3: Yes

5. Is the manuscript presented in an intelligible fashion and written in standard English?

PLOS ONE does not copyedit accepted manuscripts, so the language in submitted articles must be clear, correct, and unambiguous. Any typographical or grammatical errors should be corrected at revision, so please note any specific errors here.

Reviewer #1: Yes

Reviewer #3: Yes

6. Review Comments to the Author

Please use the space provided to explain your answers to the questions above. You may also include additional comments for the author, including concerns about dual publication, research ethics, or publication ethics. (Please upload your review as an attachment if it exceeds 20,000 characters)

Reviewer #1: It appears that the suggested editing has improved the presentation of the work herein. The supplemental data provided appear to complete requests. it appears that the manuscript is in form to be moved forward; please consider the manuscript accepted.

Reviewer #3: 1. If the authors insist doing assembly after the de-contamination, it raises the following questions:

We chose a protocol that was peer reviewed and published in PLOS ONE as well as other journals (which we detailed extensively in our previous response). If these protocols are invalid, these articles should be recalled. However it should not become our responsibility to defend peer-reviewed work that we did not author and is currently published. If the reviewer contests DeconSeq use cases, we suggest taking it up with the relevant authors.

a. is there any chimera contigs in the assembly results?

Perhaps. Other publications using Trinity and Deconseq that we have referred to did not report on chimeras. And as mentioned in previous responses, at this point we do not have the resources to address this. Moreover, there is no available genome of the African potato to compare assembled chimeras with actual chimeras.

If chimeras resemble *Helianthuus annuus* sequences more than the similar species of the African potato, they would have been removed by DeconSeq; not because they were chimeras but because they matched better to *Helianthus annuus.*

b. if yes, how many? Are these contigs been removed by the de-contamination step?

NA

c. if the chimera contigs are removed, how much data/information of the object(African potato) is lost?

NA

d. what is the effect of these data/information lose?

NA

2. is there any proof that run trinity in default parameter is acceptable on data with contaminations like yours?

Yes. We have already provided in the previous response an extensive list of cases of Trinity in conjunction DeconSeq. If the reviewer implies that there should be specific parameters for our unique case, then those other published unique cases should have underwent the same scrutiny, because they are all unique in their own right. If they are faulty, we suggest that PLOS ONE recalls those publications because they have provided misinformation to us.

7. PLOS authors have the option to publish the peer review history of their article ([what does this mean?](https://journals.plos.org/plosone/s/editorial-and-peer-review-process#loc-peer-review-history)). If published, this will include your full peer review and any attached files.


If you choose “no”, your identity will remain anonymous but your review may still be made public.


**Do you want your identity to be public for this peer review?** For information about this choice, including consent withdrawal, please see our [Privacy Policy](https://www.plos.org/privacy-policy).

Reviewer #1: **Yes:** Jian Gao

Reviewer #3: No
